# Understanding the apparent fractional charge of protons in the aqueous electrochemical double layer

**DOI:** 10.1038/s41467-018-05511-y

**Published:** 2018-08-10

**Authors:** Leanne D. Chen, Michal Bajdich, J. Mark P. Martirez, Caroline M. Krauter, Joseph A. Gauthier, Emily A. Carter, Alan C. Luntz, Karen Chan, Jens K. Nørskov

**Affiliations:** 1 SUNCAT Center for Interface Science and Catalysis, Department of Chemical Engineering, Stanford University, Stanford, CA 94305 USA; 2 SUNCAT Center for Interface Science and Catalysis, SLAC National Accelerator Laboratory, Menlo Park, CA 94025 USA; 3 Department of Mechanical and Aerospace Engineering, Princeton University, Princeton, NJ 08544 USA; 4 School of Engineering and Applied Science, Princeton University, Princeton, NJ 08544 USA; 5 Present Address: Division of Chemistry and Chemical Engineering, California Institute of Technology, Pasadena, CA 91125 USA; 6 Schrödinger GmbH, Dynamostr. 13, D-68165 Mannheim, Germany; 7 Department of Physics, Technical University of Denmark, 2800 Kongens Lyngby, Denmark

## Abstract

A detailed atomic-scale description of the electrochemical interface is essential to the understanding of electrochemical energy transformations. In this work, we investigate the charge of solvated protons at the Pt(111) | H_2_O and Al(111) | H_2_O interfaces. Using semi-local density-functional theory as well as hybrid functionals and embedded correlated wavefunction methods as higher-level benchmarks, we show that the effective charge of a solvated proton in the electrochemical double layer or outer Helmholtz plane at all levels of theory is fractional, when the solvated proton and solvent band edges are aligned correctly with the Fermi level of the metal (*E*_F_). The observed fractional charge in the absence of frontier band misalignment arises from a significant overlap between the proton and the electron density from the metal surface, and results in an energetic difference between protons in bulk solution and those in the outer Helmholtz plane.

## Abstract (web summary)

A detailed atomic-scale description of the electrochemical interface is essential to the understanding of electrochemical energy transformations. Here, the authors investigate the solvated proton at the electrochemical interface and show that it unexpectedly carries a fractional charge.

## Introduction

As the price of electricity from photovoltaics and wind turbines continues to plummet^1^, the prospects of using electrocatalysis to produce fuels and chemicals in a sustainable way become increasingly viable. Unfortunately, current state-of-the-art electrocatalysts for e.g., CO_2_ reduction are far from efficient enough to make such catalytic processes economically feasible^2^. Hence, the design of new electrocatalysts for energy transformation is a major challenge to science. To this end, a fundamental understanding of the atomic-scale processes that dictate reactivity is essential for progress toward a sustainable energy future. An accurate atomic-scale description of the interface between a solid electrode and a liquid electrolyte needs to capture simultaneously contributions from the solvent, ions in solution, and adsorbates—all under the effects of an applied electrostatic potential. This complexity, both in terms of the sheer size of the system (typically on the order of 10^2^ to 10^3^ electrons) and intricate nature of chemical and electrostatic interactions, leads to the fact that the vast majority of molecular-level theoretical descriptions of electrocatalysis is based on density-functional theory (DFT) calculations, which offer an optimal balance between accuracy and cost. Indeed, there has been a steep rise in the number of studies applying DFT calculations to electrochemical reactions in the past decade (see Supplementary Fig. 1). The question we address in the present Article is whether DFT calculations at the semi-local level of theory (the generalized gradient approximation, or GGA, commonly used in computational surface chemistry and catalysis) are accurate enough to treat the charged solid–liquid interface.

GGA-level DFT calculations have well-described issues with charge localization^3–5^. There are indications in the literature that solvated protons outside a metal surface have charges of less than unity^6–8^, which has been hypothesized to be symptomatic of the delocalization error in GGA-DFT. Moreover, the fact that the band gap in water is not properly described^9–13^ can in some cases lead to charge transfer to or from the water layer either in the bulk for solutes^14–16^, or when solvent or solvated ion band edges cross the metal’s Fermi level ($E_{\mathrm{F}}$)^17,18^. It is well-known that partial charge transfer occurs between the adsorbate and the electrode in an electrochemical environment^19^; the focus of the present Article is on the partial charge transfer between a solvated species in the outer Helmholtz layer and the electrode. Solvated protons are essential components in many energy-related electrocatalytic reactions (e.g., water splitting, hydrogen evolution, CO_2_ reduction, and N_2_ reduction). Hence, understanding the differences between a proton in its bulk solvation environment and when the proton has diffused to the electrochemical double layer immediately prior to the proton-coupled electron transfer is of prime importance. Herein, we apply DFT calculations both at and beyond the GGA level to a static model of the protonated Pt(111) | H_2_O and Al(111) | H_2_O interfaces to examine specifically the charges of protons in the electrochemical double layer. We show that in all cases where GGA-DFT gives the correct band alignment between the metal system and acidified water, the description of the charge is comparable to hybrid and embedded correlated wavefunction methods within 0.1 *e* precision. The agreement between GGA-DFT and higher-level methods suggests that the fractional nature of the proton solvated in the electrochemical double layer is a physical phenomenon.

## Results

In the following, we first show that for protons solvated in the first water layer (i.e., outer Helmholtz plane) outside a Pt(111) surface, the net charge is close to +0.7 *e* per proton calculated with all levels of theory, whereas the net charge approaches +1.0 *e* when solvated in the third water layer and no longer in close proximity to the electrode. We note that calculations have also been carried out with a proton solvated in the second water layer, whose charge agrees with that of a proton in the third solvent layer, indicating that these charges have approached a converged bulk value. We then go on to show the origin of the surprising fractional charge for the solvated proton in the outer Helmholtz plane to be the extensive overlap between the metallic and nearest solvent layer electron densities.

### The Pt(111) | H_2_O interface

**Fig. 1:**
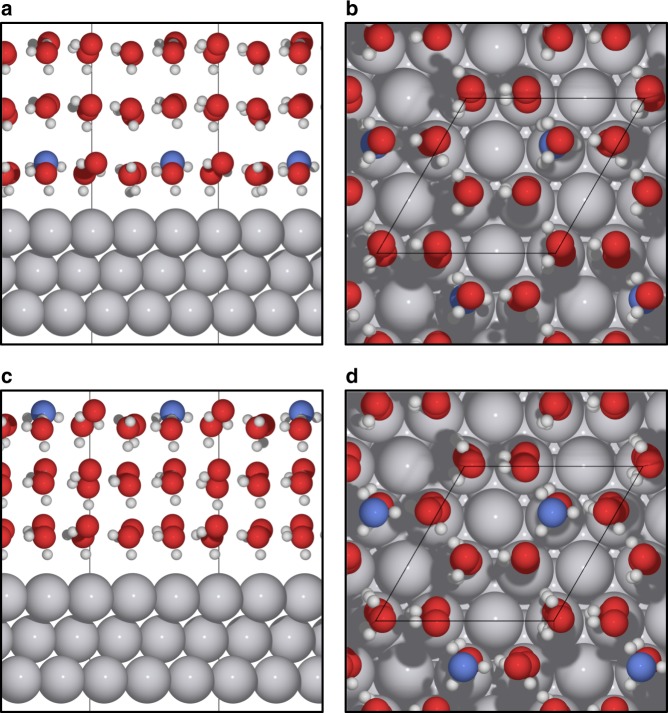
Computational cells for the Pt(111) | acidified water interface. Gray spheres represent Pt atoms, red spheres represent O atoms, and white spheres represent H atoms. The hydronium oxygen is highlighted in blue in all cases. Periodic cells are separated by at least 15 Å of vacuum in the *z* direction between the metal and the solvent. **a**, **b** Side and top views of a proton in the outer Helmholtz plane, respectively. **c**, **d** Side and top views of a proton in the third solvent layer, respectively

Many different approaches have been used to model the double layer owing to its importance: from classical continuum treatments^20–24^, counter-electrodes^25^, discrete counter-charges^26,27^, to explicit solvated ions and water layers to tune the potential^28–30^. A number of studies have also combined implicit and explicit solvent descriptions to specifically examine electrochemical reactions^31–34^. In this study, our model for the electrochemical double layer is the Pt(111) | acidified water interface with three layers of metal and three layers of solvent. Two example structures are illustrated in Fig. 1. The geometry is based on hexagonal ice-like water structures that have been observed both experimentally at low temperatures under ultra-high vacuum conditions^35^ as well as in DFT simulations^36^. We note that a real electrochemical system is typically run at room temperature where the water structure is more disordered as suggested by ab initio molecular dynamics simulations^37–39^. Since the focus of our study is on assessing the accuracy of charge determination with higher-level benchmarks, we use the well-studied ice-like ideal structure as our system of interest to explore potential GGA-DFT errors. Moreover, we have examined a number of water structures and observed that the charge on the proton does not vary with changing geometry or proton concentration in the double layer (see Supplementary Fig. 2).

The proton is simulated by adding an extra H atom to the solvent, which separates into an electron on the metal surface and a proton in solution upon optimization of the electronic density. The excess negative charge at the surface represents the electrode under a negative potential bias, or a reducing environment. We examine two situations: one where the proton is in the first solvent layer adjacent to the electrode (the outer Helmholtz plane)—which is the relevant environment for intermediates in electrocatalytic reactions, and another where the proton is spatially separated from the electrode (the third solvent layer).

**Fig. 2:**
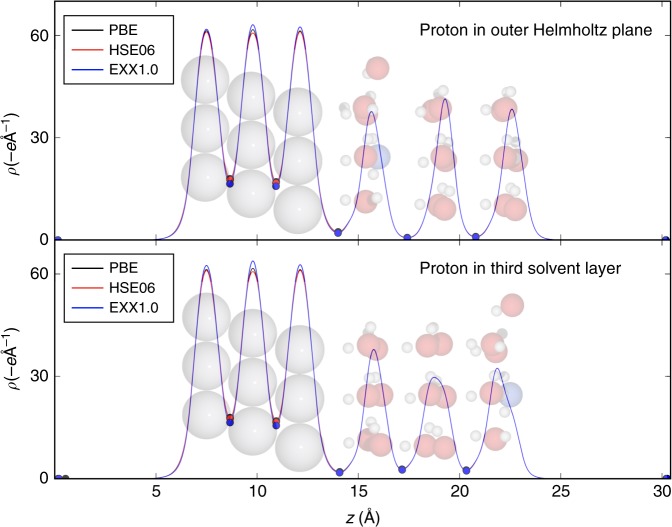
Electron density analysis for the solvated proton. Plane-integrated electron density distribution as a function of the unit cell *z*-coordinate calculated with PBE (black), HSE06 (red), and EXX1.0 (blue) exchange-correlation functionals. The top and bottom panels show H_3_O^+^ in the outer Helmholtz plane (first solvent layer) and third solvent layer, respectively. The plane-integrated charge for the solvated proton is fractional in the first solvent layer with values of +0.64, +0.69, and +0.71 *e* at the PBE, HSE06, and EXX1.0 levels of theory. These charges approach unity with values of +0.86, +0.88, and +0.91 *e*, respectively, for the PBE, HSE06, and EXX1.0 functionals where the proton is in the third solvent layer. The charges on the proton obtained for each structure with all three functionals agree within 0.1 *e*

To determine the charge of a proton solvated in the outer Helmholtz plane, we apply DFT calculations at three different levels of theory (PBE, HSE06, and EXX1.0) to the Pt(111) | acidified H_2_O interface. PBE^40,41^ is a semi-local functional widely used in surface chemistry, HSE06^42^ is a hybrid functional that corrects a portion of the charge delocalization error inherent to semi-local functionals by including a fraction (0.25) of the Hartree–Fock or exact exchange (EXX), and EXX1.0 is a modified version of HSE06 that has been shown to describe charge localization well in molecular systems (see Supplementary Fig. 3), where we include the full (1.0) exact exchange and therefore it serves as one of our higher-accuracy benchmarks. Although the use of EXX can be problematic in metals, it greatly improves the description of the electronic structure of water (for a more thorough description of these functionals, see the Methods section). Figure 2 shows the *xy* plane-integrated electron densities for the Pt(111) | protonated water system at the PBE (black), HSE06 (red), and EXX1.0 (blue) levels of theory with minima positions indicated as filled circles. The top panel shows the plane-integrated electron density for a proton solvated in the outer Helmholtz plane for the Pt(111) | H_2_O interface, and the bottom panel shows the corresponding electron density for a proton in the third solvent layer for the same interface. Here we use a simple partitioning scheme equivalent to Bader analysis^43,44^ in a single dimension: the *xy* plane-integrated electron densities are plotted as a function of *z*, which is the direction perpendicular to the surface. To accurately determine the minima of the resulting 1D density, the finite mesh values are interpolated with a spline function, which is then used to search analytically for local minima. To obtain the charge in the metal, this 1D electron density is integrated from a *z* value of 0 (on the vacuum side of the slab) to the electron density minimum separating the slab and the solvent. The charge of the proton in the outer Helmholtz plane is determined by integrating the electron density between the slab and the first water layer, then subtracting from it the electron density of a neutral water layer. For the proton in the third solvent layer, the integration limits are at the minimum between the first and second water layers and the upper limit of the cell (*z* = 30.4 Å). Note that Bader analysis in three dimensions on the solvated proton gives the same results (see Supplementary Table 1). The reason that we have used this simplified partitioning scheme is that in a capacitor model developed for electrochemical reactions^7,8^, the potential dependence is only a function of the distance of the charged species from the surface (the *z* direction). Moreover, the excess charge is only slightly delocalized to the other water molecules within the same layer (0.11 *e*) with the majority of the positive charge residing on the hydronium ion itself (see Supplementary Fig. 4), and collapsing the *x* and *y* directions affords a clearer picture of the charge separation at the electrochemical double layer. The charge computed from the capacitor model dictates the variation of energy vs. the work function of the interface (see, for example, Supplementary Fig. 2) and provides the potential dependence of the energetics of charge transfer reactions across the interface.

Figure 2 shows that the plane-integrated charge for the solvated proton is clearly fractional in the outer Helmholtz plane with values of +0.64, +0.69, and +0.71 *e* at the PBE, HSE06, and EXX1.0 levels of theory. However, in the case of the proton in the third solvent layer, our calculated charges approach unity (+0.86, +0.88, and +0.91 *e*, respectively, for PBE, HSE06, and EXX1.0). We note that the determination of these charges has an uncertainty of 0.1 *e* as discussed in Supplementary Note 1, which is likely why we do not obtain a charge of exactly +1.0 *e* in the third solvent layer for the proton.

**Fig. 3:**
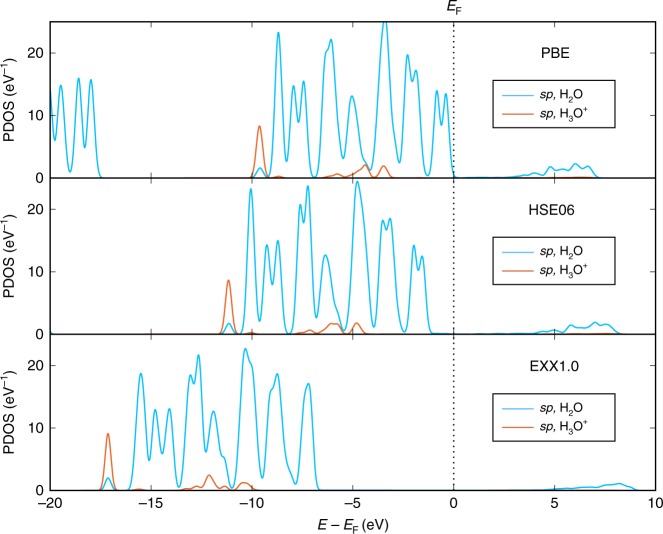
Projected solvent and ion density of states for the Pt(111) | acidified water interface. The proton is solvated in the outer Helmholtz plane as shown in Fig. 1a and b. The geometry is optimized at the PBE-DFT level of theory. Three different methods are shown for the proton, from top to bottom: PBE, HSE06, and EXX1.0. The valence states (2*s*, 2*p*) are projected for oxygen and the 1*s* states are projected for hydrogen

In Fig. 3, we show the projected density of states for water molecules at the Pt(111) | acidified water interface, calculated with the PBE, HSE06, EXX1.0 exchange-correlation functionals (top to bottom panels, in order). In all cases except PBE, the highest occupied molecular orbital (HOMO) and the lowest unoccupied molecular orbital (LUMO) of both the solvent and the proton clearly straddle the Fermi level of the metal, which ensures that there is no artificial charge transfer between the solvent and metal. The result of the PBE functional is a borderline case, since the HOMO of solvent terminates directly at the Fermi level. The charges on the hydronium obtained with all three levels of theory agree within 0.1 *e* (the precision threshold for our charge partitioning scheme), which suggests that the solvent and metal bands are correctly aligned in our double layer structure. In general, such a band misalignment results from a combination of the underestimation of the band gap of water in GGA-level functionals as well as the relative position of *E*_F_ with respect to the vacuum level, which can be shifted by modifying the surface dipole using different solvation structures^7,8,17,37,45,46^.

### The Al(111) | H_2_O interface

**Fig. 4:**
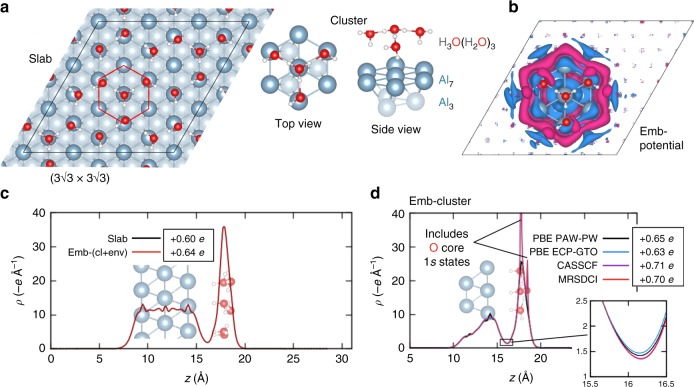
Embedded correlated wavefunction models and predictions for solvated protons near an Al(111) surface. **a** Slab: $(3\sqrt 3 ) \times 3\sqrt 3$)*R*30° three-layer Al(111) + (H_2_O)_15_(H_3_O)_3_, and cluster: Al_10_(H_2_O)_3_H_3_O, models for the solvated hydronium on Al. **b** Embedding potential optimized for the slab and cluster described in **a** (blue and magenta isosurfaces correspond to +1.5 and −1.5 V, respectively). **c** Plane-integrated GGA-DFT valence electron density distributions (renormalized per H_3_O^+^) along the surface normal for the full slab (black) and the sum of the embedded cluster and environment (red). **d** Comparison of electron density distribution from embedded cluster PBE-DFT (planewave, PW, and Gaussian-type orbital, GTO), complete active space self-consistent field (CASSCF) (11e,10o), and multi-reference singles and doubles configuration interaction (MRSDCI) methods. The inset highlights the position of the minimum of the distribution at the metal-solvent interface from different methods. In both **c** and **d**, the net charge of the solvent layer (from the minimum at ~16.2 Å) is shown to be fractional

We also perform benchmark calculations of the solvated proton charge using correlated wavefunction (CW) methods. Because Al has a significantly lower work function than Pt (4.2 vs. 5.7 eV for the (111) surfaces) and thus represents a more electron-donating surface^47^, it provides a more rigourous test of whether CW methods can predict a solvated proton in the outer Helmholtz plane to have a well-defined +1.0 *e* charge. An Al(111) slab with an effective surface coverage of 1 H_3_O^+^ per (3 × 3) supercell is chosen as a model (see Fig. 4a). To manage the computational cost, density-functional embedding theory (DFET) developed by Huang, Pavone, and Carter^48^ was employed. Using this method, a smaller fragment of the total system can be modeled using a higher-level CW method, while the correct boundary conditions are imposed around the fragment by means of an effective embedding potential. Within the DFET formalism, the embedding potential represents the interaction potential between the fragments obtained via DFT (PBE, in the current discussion). Figure 4a shows the structural partitioning employed, where an Al_10_ cluster and a single H_3_O^+^ ion surrounded by three H_2_O molecules represent the region of interest, while Fig. 4b shows the optimized embedding potential derived from PBE-DFT (see the Methods section for more details). The negative embedding potential surrounding the cluster serves to delocalize the electron density in the region between the cluster and environment to account for the metallic bonds between the fragments (see also Supplementary Fig. 5). Figure 4c shows the plane-integrated valence electron density plots for the slab and the sum of the densities from the embedded cluster and environment as functions of the *z*-coordinate. Optimization of the embedding potential enforces the densities of the aforementioned total system and sum of subsystems to be approximately equal. The residual charges in the outer Helmholtz plane are in agreement to within 0.04 *e*. Using the same optimized embedding potential (embedded PBE-DFT), complete active space self-consistent field (CASSCF)^49–51^ with 11 electrons and 10 orbitals in the active space—i.e., CAS (11e,10o)—and multi-reference singles and doubles configuration interaction (MRSDCI)^52^ from CAS (11e,10o) were calculated at an augmented double-*ζ* basis set level using a Gaussian-type orbital (GTO) basis and an effective core potential (ECP) for Al (more details can be found in the Methods section). We chose these CW methods to accurately capture the multi-configurational nature of metals, which is lacking in single reference methods (e.g., Møller–Plesset second order perturbation theory (MP2) and most of the current coupled-cluster implementations). Additionally, these embedded-CW methods have been shown to correctly predict physical charge transfer behavior between molecules and metallic surfaces^53–56^. Figure 4d shows the corresponding electron density plots from the previously discussed methods, with the PBE-DFT planewave (PW) projector augmented wave (PAW) result also shown for comparison. Because of the all-electron treatment of O in the GTO basis set calculations, the core 1*s* electrons of O are also plotted, leading to double peaks within the solvent layer. The residual charges in the solvent layer, calculated by integrating the electron density from the minimum between the metal and the solvent (~16.2 Å) up to ~6.5 Å out into the vacuum (where the plane-integrated electron density decays to ~10^−5^ *e*/Å), are also shown. Note that this residual charge depends on the basis set and core representation used (and real-space grid used to represent the charges, see Supplementary Note 2); thus, for a more fair comparison, the charges must be compared within the same basis set and grid density. Noticeably, the CW methods CASSCF and MRSDCI (which use the full exact exchange) predict higher residual charges than PBE-DFT, albeit only by 0.1 *e*. This is consistent with the result from the hybrid functionals in the previous sections. Our analyses of both the Pt | water and Al | water interfaces at multiple levels of theory together show the fractional charge of a proton in the outer Helmholtz plane to be a physical phenomenon and not simply an artifact of DFT-GGA self-interaction error.

### Origin of the fractional charge

**Fig. 5:**
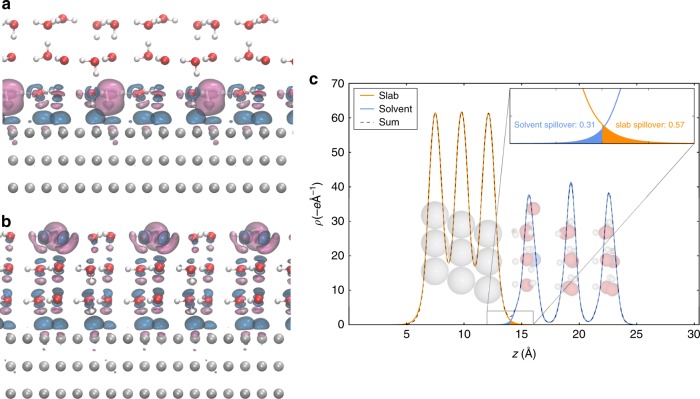
Explanation for the effective fractional charge of a proton in the outer Helmholtz plane. **a**, **b** Electron density difference plot for the solvated proton in the outer Helmholtz plane and third solvent layer (respectively) obtained at the PBE-DFT level. Isosurfaces of –0.02 *e* Å^−3^ (electron accumulation) and 0.02 *e* Å^−3^ (electron depletion) are shown in blue and magenta, respectively. **c**, Plane-integrated electron density distribution as a function of the unit cell *z*-coordinate calculated separately for the negatively charged slab (orange) and positively charged solvent (blue) (see the main text for further details on these calculations). The excess charge from the slab (solvent) is integrated in the first layer solvent (slab) region to quantify the extent of the electron spillover in their complementary regions. The net charge on the proton is +0.74 *e* from this analysis, in agreement with the raw charges extracted from calculations on the total system. The black dashed lines represent the total plane-integrated electron density of the system as a reference

In what follows, we show that the fractional charge observed for protons in the outer Helmholtz plane originates from the overlap of electron density of the metal electrode and that of the charged solvent. Figure 5a and b shows the electron density difference isosurfaces for the Pt(111) | acidified water system. This electron density difference is given by subtracting the electron density of individual neutral components from that of the interacting system,

1 $${\mathrm{\Delta }}\rho = \rho _{{\mathrm{all}}} - \rho _{{\mathrm{metal}}} - \rho _{{\mathrm{solvent}}} - \rho _{\mathrm{H}}$$

which gives the charge redistribution of the system upon interaction of the fragments. In both cases, there is excess electron density immediately outside the Pt slab, which has some overlap with the proton in the first layer of solvent molecules and, as a result, the net assigned charge is positive and fractional (Fig. 5a). As we take the hydronium into the third solvent layer (Fig. 5b), it no longer has direct overlap with the surface charge spillover and therefore its charge approaches an integral value of +1.0 *e*.

To quantify the degree of electron spillover, we calculated the electron densities of a negatively charged slab and positively charged solvent separately at the PBE-DFT level, which provide the charges of the two fragments at the limit of complete separation. For the negatively charged slab (orange trace in Fig. 5c), the positive counter charge is provided by a linearized Poisson–Boltzmann distribution^23^ outside the surface to ensure overall neutrality of the simulation cell. For the positively charged solvent (blue trace in Fig. 5c), a negative homogeneous background charge of unity is sufficient to set up the simulation. Adding up the two partial densities gives the total electron density (dashed black trace in Fig. 5c), which is identical to what we extract normally from a calculation on the total system. In Fig. 5c, we see that both the solvent and the slab have a spillover of electron density into the complementary region at the interface. The spillover from the negatively charged slab into the solvent is 0.57 electrons, and the spillover from the positively charged solvent into the slab is 0.31 electrons. This results in a net spillover of 0.26 electrons into the first layer of solvent, and is in fact what gives rise to the fractional charge of the proton in the outer Helmholtz plane. The net charge on the proton from the analysis in Fig. 5c is therefore +0.74 *e*, in good agreement with the charge partitioning results in Fig. 2 at all three levels of theory. This tail of electron density from the slab does not extend beyond the first water layer, which is why we are able to recover the correct charge (aside from normal charge partitioning variations) within 0.1 *e* for the proton in the third solvent layer. Therefore, even when forcefully partitioning the electrons in order to facilitate realization of fully ionized fragments, the largest effective charge that can be achieved for a proton in the outer Helmholtz plane remains close to the predicted values of methods that completely remove the self-interaction error in the interacting system.

In summary, we have demonstrated that protons in the outer Helmholtz plane of the electrochemical interface have a physical fractional charge utilizing both hybrid-DFT and embedded correlated wavefunction methods. The fractional charge arises from the overlap of electron density from both the metal and solvent components. This picture is consistent with that obtained with a simple GGA-level description of the electrochemical interface, which suggests that electrochemical charge transfer reactions are sufficiently well-described using GGA functionals when the ion and solvent band edges are aligned correctly with *E*_F_.

This fractional charge of protons in the outer Helmholtz plane has major implications for electrochemical kinetics, since the partial discharge of protons upon approaching the electrode surface would give rise to a potential dependence in the reaction energetics in proton-coupled electron transfers (PCETs). Whereas the initial state in the computational hydrogen electrode method^57^ involves a proton solvated in bulk liquid, the electron density overlap detailed in this study suggests that a proton in the outer Helmholtz plane cannot be assumed to be at the same energy level as the protons in bulk solution. Moreover, concentration effects related to the availability of proton donors at the surface would also come into play in kinetic analyses. Future work will bridge the bulk and interface paradigms by addressing this charge-potential relation at the outer Helmholtz plane and its specific effects on the energetics of PCETs at a charged solid–liquid interface.

## Methods

### DFT calculation details

All periodic electronic structure calculations were carried out with the Vienna Ab initio Simulation Package (VASP)^58–61^. (3 × 3) supercells of the FCC(111) unit cell were used for both Pt and Al with three solvent layers. Each solvent layer contains eight water molecules. Three metallic layers were used to simulate the Pt(111) and Al(111) slabs with the two bottom layers fixed at the bulk Pt/Al lattice constants. All other atoms were allowed to relax until all forces were below 0.05 eV/Å. Periodic images were separated by at least 15 Å of vacuum in the *z* direction and the dipole correction^62^ was used to decouple the electrostatic interaction between periodically repeated slabs. Geometries were optimized with a (4 × 4 × 1) Γ-centered *k*-point mesh^63^ using a planewave basis kinetic energy cutoff of 400 eV with ultrasoft pseudopotentials^64^ at the PBE-DFT level of theory^40^. Single-point calculations using 0.25 (HSE06)^42^ and 1.0 (EXX1.0) short-range Hartree–Fock exchange with a reduced screening length of 0.1/Å were performed on the PBE-optimized geometries to examine whether the electron density changes upon localizing the electrons. A reduction in the screening length from the HSE06 default of 0.2 to 0.1/Å was employed in order to reduce the missing fraction of the charge on the proton.

### Embedded DFT and CW calculation details

The embedded spin-restricted DFT, CASSCF, and MRSDCI densities were calculated using MOLPRO^65^. The embedding potential applied to the calculations was determined at the DFT level with the PBE functional as described in Supplementary Note 3. This embedding potential is then used to include the effect of the environment for the embedded cluster calculations by adding it to the Hamiltonian via the matrix manipulation feature in MOLPRO. An open-source code developed in the Carter group was used to construct the embedding potential in the atomic orbital basis from a real-space grid basis^66^. The basis set for the Al atoms was based on the Hay-Wadt(2*s*, 2*p*) LANL2DZ^67^ 10-electron effective core potential (ECP) and basis set. We added a polarization function with exponent (0.19 a.u.)^68^, and a set of diffuse (*s*, *p*, *d*) functions with exponents (0.0237, 0.0167, 0.04 a.u.) in an even-tempered way. For H and O atoms, Dunning’s augmented double-*ζ* basis set aug-cc-pvdz^69,70^ was used. For all cases, the *S* = ${\textstyle{1 \over 2}}$ spin state was calculated, and in all the methods, it was found that the unpaired electron is strongly localized in the Al cluster. For CASSCF calculations, an active space of 11 electrons in 10 orbitals was selected from the DFT orbitals around the Fermi level as the initial guess (a calculation with an active space of 15 electrons in 14 orbitals confirmed convergence with respect to size of the active space; DFT instead of Hartree–Fock orbitals were used as starting guess because the former are usually better adapted for metallic systems) and all the orbitals were relaxed throughout the course of the calculations (see Supplementary Fig. 6 for the converged CAS natural orbitals). During the subsequent MRSDCI calculations, the energetically lowest eight orbitals were frozen (no excitations considered from these orbitals) and the reference space was constructed from all CASSCF configurations with an absolute coefficient of >0.05.

### Data availability

The data that support the findings of this study are available from the corresponding author on reasonable request.

## Electronic supplementary material

Supplementary Information
