# Supplementary material for: Understanding the apparent fractional charge of protons in the aqueous electrochemical double layer
Source: Nat Commun. 2018 Aug 10;9:3202. doi: 10.1038/s41467-018-05511-y (PMC6086897; doi:10.1038/s41467-018-05511-y)
Supplement: Supplementary file 1 — Supplementary Information [file 41467_2018_5511_MOESM1_ESM.pdf]

Supplementary Information for

Understanding the apparent fractional charge of protons in the aqueous electrochemical double layer

Chen et al.

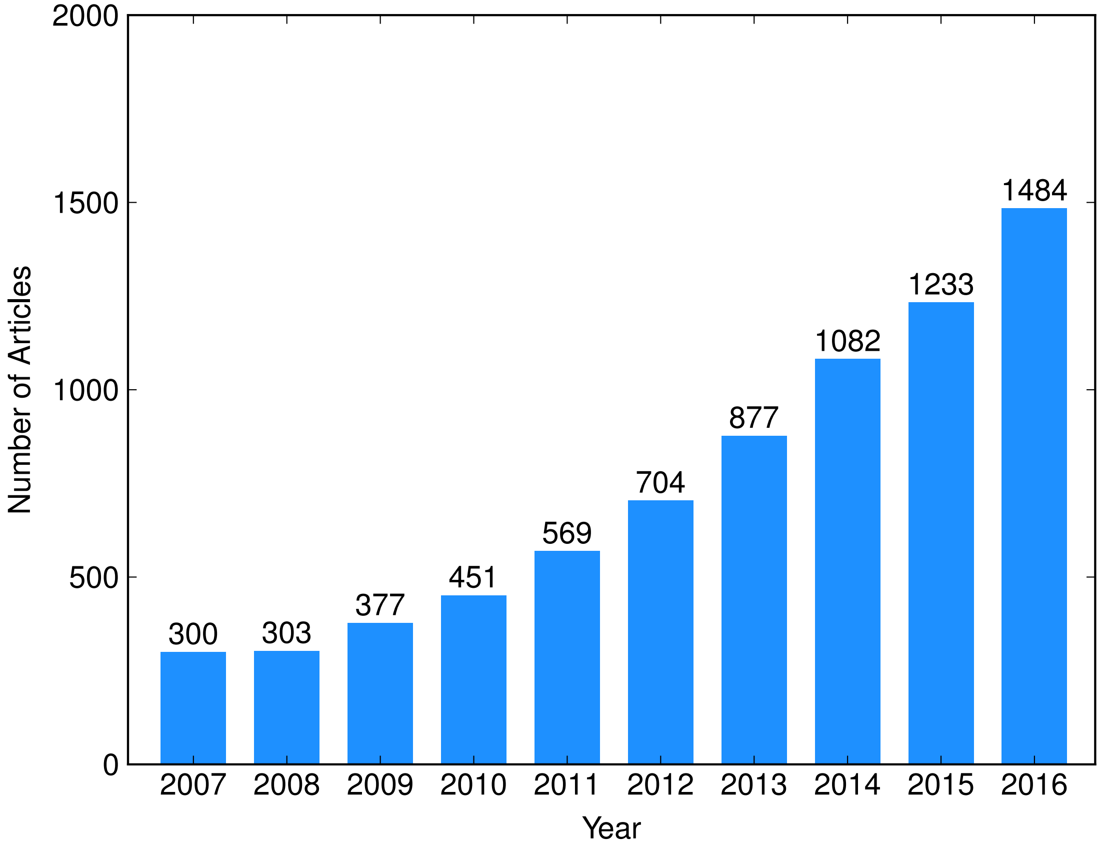

Supplementary Figure 1 | Web of Science search of electrochemical reactions studied with density functional theory. These data were collected using the Web of Science search engine for articles containing both the phrases "density functional theory"/DFT and "electrochemi*", where the quotes enclose exact-phrase matching and the asterisk indicates an any-character-length wildcard.

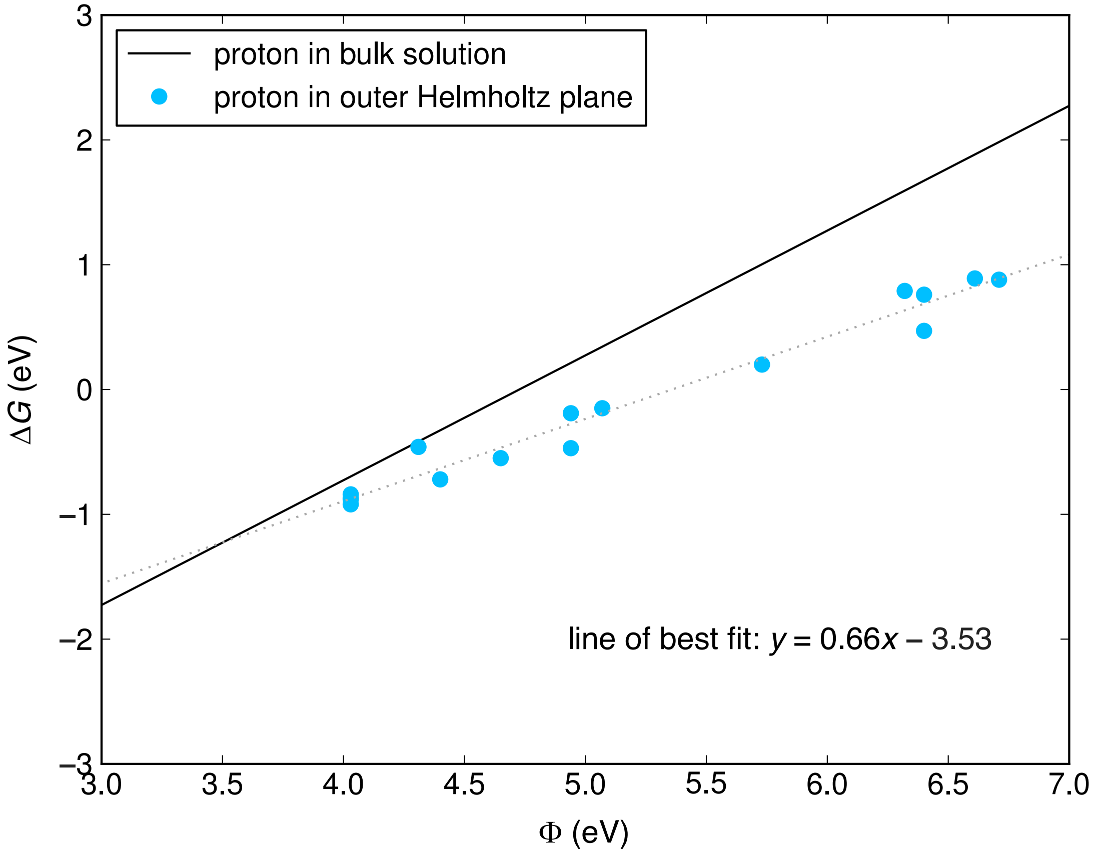

Supplementary Figure 2 | Reaction energies for the Volmer step on Pt(111) under acidic conditions calculated with different water structures. The Volmer reaction involves a proton-electron pair adsorbing to an empty site on a metal surface, written as $H^+ + e^- + * \rightleftharpoons H^*$. The Volmer reaction energy is shown at different work functions on a Pt(111) surface, obtained with different cell sizes and water structures.[1] The dependence of the reaction energy on potential should be unity, corresponding to the expected charge of a solvated hydronium ion, as shown in the black trace (the computational hydrogen electrode reference).[2] However, explicitly simulating the proton in the outer Helmholtz layer gives a charge of between 0.6 to 0.7, as shown by the slope of the line of best fit for the blue data points.

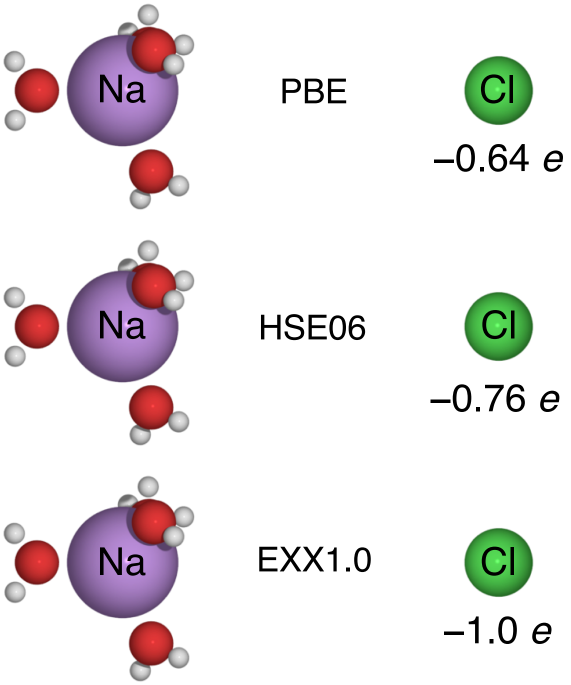

Supplementary Figure 3 | Charge on a NaCl molecule separated at 10 Å obtained at different levels of theory. The correct integral charge on chloride is obtained only with EXX1.0. The sodium ion has a charge of +0.99 in all cases, with the residual charges localizing on the water molecules. The residual charges on the water molecules are omitted for clarity.

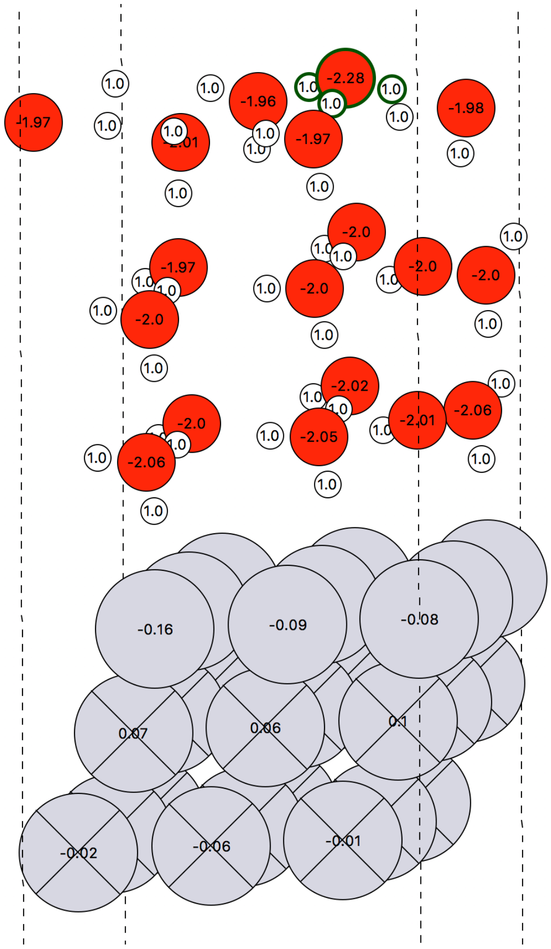

Supplementary Figure 4 | Atom-decomposed Bader charges for the 3×3 cell with the proton solvated in the outermost water layer. The hydronium ion is highlighted in bold outlines, and the net charge is shown for each atom (relative to the neutral atom). The excess charge is only slightly delocalized to the other water molecules within the same layer (0.11 *e*), with the majority of the positive charge residing on the hydronium ion itself.

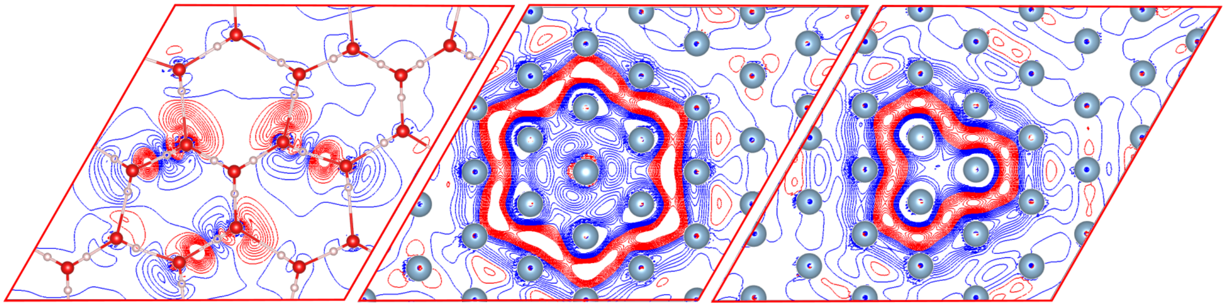

Supplementary Figure 5 | Contour plots showing the embedding potential at particular planar slices cutting through the solvent layer and the Al slab. Red: negative, blue: positive, contour line intervals: 0.2 V (set minimum: –2 V, set maximum: +2 V). The negative potentials between the O and H atoms of the water molecules that form hydrogen bonds in the full system, serve to simulate these severed interactions (left panel). Slices through the Al slab (center and right panels) show the sharper negative potential features between the Al atoms at the cluster-environment interface to simulate metallic bonding. See also Figure 4a and 4b in the main text for structural legends.

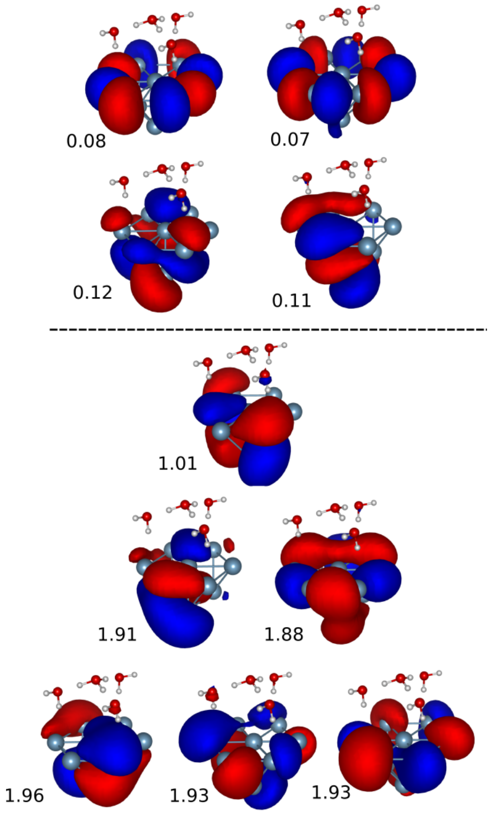

Supplementary Figure 6 | CAS natural orbitals and their respective occupations for the CAS (11e,10o) obtained from the embedded-CASSCF calculation for the $Al_{10}(H_2O)_3(H_3O)$ cluster.

Al: blue spheres, O: red spheres, and H: grey spheres. Isosurface: 0.02 a.u.

Supplementary Table 1 | Comparison of Charge Partitioning Schemes for H_3O^+ in the third solvent layer.

Scheme	Metal	Solvent 1	Solvent 2	Solvent 3	Charge
Ideal	271.00	48.00	48.00	48.00	+1.00
Bader	270.66	48.20	47.97	48.17	+0.86
Minima	270.66	48.20	47.97	48.17	+0.86
Average $\Delta\rho = 0$	270.99	47.85	48.01	48.15	+0.84
Discrete $\Delta\rho = 0$	270.86	48.03	47.99	48.12	+0.89

Supplementary Note 1 | Charge Partitioning Schemes

We analyzed the charge in the metal and solvent using several partitioning methods, all of which gave charges similar to within 0.1 *e*. These charge partitioning schemes with a proton in the third solvent layer are summarized in Supplementary Table 1. The number of electrons in each layer is listed for each method of partitioning, with the expected (column "Ideal") numbers listed for reference. These partitioning schemes used the electron density obtained with the PBE functional.[3] The charge of the ion is obtained by taking the outer two solvent layers and subtracting from it the number of electrons in two neutral water layers (96). Row "Bader" was obtained with Bader charge analysis.[4,5] Row "Minima" was calculated by finding local minima in the plane-integrated total electron density and integrating between these minima. The integration limits in row "Average $\Delta\rho$" was obtained by first taking the electron density difference between the system and its individual components:

$$\Delta\rho = \rho_{\mathrm{all}} - \rho_{\mathrm{metal}} - \rho_{\mathrm{solvent}} - \rho_{\mathrm{ion}} \tag{1}$$

then identifying nodes in the resulting plane-integrated electron density difference plots. Finally, "Discrete $\Delta\rho = 0$" takes individual nodal points in the electron density differences and integrates between them, as opposed to the plane-integrated electron density differences which has nodal planes. Note that all integration is done on the total valence electron density. All four charge partitioning methods give values that vary within 0.1 *e*. Thus, we use the Minima method throughout the main text due to its consistency with Bader analysis and physical simplicity.

Supplementary Note 2 | Charge Integration in Embedded DFT and CW Methods.

To integrate the electron density when using a GTO basis, a ($280 \times 280 \times 900$) three-dimensional Cartesian grid of step size 0.1, 0.1, and 0.04 Bohr along x, y, and z is used to represent the electron density in real space [total volume = ($28 \times 28 \times 36$) Bohr^3]. To calculate for the residual charge in the solvent layer, the minima of the plane-integrated electron densities between the metal and the solvent layer are numerically determined; from this point, the plane-integrated electron densities are numerically integrated along z via the the trapezoidal rule. Here, the fine grid density is required to be able to properly accommodate the real-space integration of the sharp features at the core regions within the solvent layers arising from the O 1s states.

Supplementary Note 3 | Optimization of the Embedding Potential from DFET

From the relaxed structure obtained using the procedure described in the main text for a three-layer (3×3) Al(111)+$(H_2O)_5(H_3O)$ slab and vacuum size of $\approx$ 20 Å, a larger three-layer slab of size $(3\sqrt{3}) \times 3\sqrt{3})R30^\circ$ Al(111)+$(H_2O)_{15}(H_3O)_3$ was constructed. The electron density for the larger slab was then re-optimized at a 660 eV plane-wave kinetic energy cut-off with a (3×3×1) Γ-centered k-point mesh. Electronic smearing of 0.09 eV introduced via the Methfessel-Paxton method[6] was used to aid electronic convergence. Additionally, a dipole correction to the potential and energy is employed. A fragment corresponding to $Al_{10}(H_2O)_3H_3O$ was assigned as the cluster, while the rest of the atoms $[Al_{71}(H_2O)_{12}(H_3O)_2]$ were treated as the environment. The embedding potential within the PAW formalism was optimized using an in-house modified VASP code version 5.3.3.[7] For the cluster, only the Γ point is sampled in the Brillouin zone. The optimized embedding potential has a root-mean-square deviation on the sum of the fragment electron densities relative to the full slab of about 4×10^{-5} e/Å^3.

Supplementary References

1. Chan, K. & Nørskov, J. K. Electrochemical barriers made simple. *J. Phys. Chem. Lett.* **6**, 2663–2668 (2015).

2. Nørskov, J. K. *et al.* Origin of the overpotential for oxygen reduction at a fuel-cell cathode. *J. Phys. Chem. B* **108**, 17886–17892 (2004).

3. Perdew, J. P., Burke, K. & Ernzerhof, M. Generalized gradient approximation made simple. *Phys. Rev. Lett.* **77**, 3865–3868 (1996).

4. Henkelman, G., Arnaldsson, A. & Jónsson, H. A fast and robust algorithm for Bader decomposition of charge density. *Comput. Mater. Sci.* **36**, 354–360 (2006).

5. Tang, W., Sanville, E. & Henkelman, G. A grid-based Bader analysis algorithm without lattice bias. *J. Phys.: Condens. Matter* **21**, 084204 (2009).

6. Methfessel, M. & Paxton, A. T. High-precision sampling for Brillouin-zone integration in metals. *Phys. Rev. B* **40**, 3616–3621 (1989).

7. Yu, K., Libisch, F. & Carter, E. A. Implementation of density functional embedding theory within the projector-augmented-wave method and applications to semiconductor defect states. *J. Chem. Phys.* **143**, 102806 (2015).
